# C10orf99/GPR15L Regulates Proinflammatory Response of Keratinocytes and Barrier Formation of the Skin

**DOI:** 10.3389/fimmu.2022.825032

**Published:** 2022-02-22

**Authors:** Teruki Dainichi, Yuri Nakano, Hiromi Doi, Satoshi Nakamizo, Saeko Nakajima, Reiko Matsumoto, Thomas Farkas, Pui Mun Wong, Vipin Narang, Ricardo Moreno Traspas, Eiryo Kawakami, Emma Guttman-Yassky, Oliver Dreesen, Thomas Litman, Bruno Reversade, Kenji Kabashima

**Affiliations:** ^1^ Department of Dermatology, Faculty of Medicine, Kagawa University, Miki-cho, Japan; ^2^ Department of Dermatology, Graduate School of Medicine, Kyoto University, Kyoto, Japan; ^3^ Agency for Science, Technology and Research (A*STAR) Skin Research Laboratories (A*SRL), A*STAR, Biopolis, Singapore, Singapore; ^4^ Department of Drug Discovery for Inflammatory Skin Diseases, Graduate School of Medicine, Kyoto University, Kyoto, Japan; ^5^ Department of Immunology and Microbiology, University of Copenhagen, Copenhagen, Denmark; ^6^ Genome Institute of Singapore (GIS), Agency for Science, Technology and Research (A*STAR), Biopolis, Singapore, Singapore; ^7^ Singapore Immunology Network (SIgN), Agency for Science, Technology and Research (A*STAR), Biopolis, Singapore, Singapore; ^8^ Advanced Data Science Project (ADSP), RIKEN, Yokohama, Japan; ^9^ Artificial Intelligence Medicine, Graduate School of Medicine, Chiba University, Chiba, Japan; ^10^ Department of Dermatology, Icahn School of Medicine at Mount Sinai, New York, NY, United States

**Keywords:** EIME, keratinocyte, GPR15L, C10orf99, 2610528A11Rik, atopic dermatitis, psoriasis

## Abstract

The epidermis, outermost layer of the skin, forms a barrier and is involved in innate and adaptive immunity in an organism. Keratinocytes participate in all these three protective processes. However, a regulator of keratinocyte protective responses against external dangers and stresses remains elusive. We found that upregulation of the orphan gene *2610528A11Rik* was a common factor in the skin of mice with several types of inflammation. In the human epidermis, peptide expression of G protein-coupled receptor 15 ligand (GPR15L), encoded by the human ortholog *C10orf99*, was highly induced in the lesional skin of patients with atopic dermatitis or psoriasis. *C10orf99* gene transfection into normal human epidermal keratinocytes (NHEKs) induced the expression of inflammatory mediators and reduced the expression of barrier-related genes. Gene ontology analyses showed its association with translation, mitogen-activated protein kinase (MAPK), mitochondria, and lipid metabolism. Treatment with GPR15L reduced the expression levels of filaggrin and loricrin in human keratinocyte 3D cultures. Instead, their expression levels in mouse primary cultured keratinocytes did not show significant differences between the wild-type and *2610528A11Rik* deficient keratinocytes. Lipopolysaccharide-induced expression of *Il1b* and *Il6* was less in *2610528A11Rik* deficient mouse keratinocytes than in wild-type, and imiquimod-induced psoriatic dermatitis was blunted in *2610528A11Rik* deficient mice. Furthermore, repetitive subcutaneous injection of GPR15L in mouse ears induced skin inflammation in a dose-dependent manner. These results suggest that C10orf99/GPR15L is a primary inducible regulator that reduces the barrier formation and induces the inflammatory response of keratinocytes.

## Introduction

The defense system of the skin has three layers: barrier, innate immunity, and acquired immunity, and is organized to elicit effective responses to protect an organism from external dangers and stresses (1). The barrier is a constitutive machinery at the outermost sites of the organism physically, chemically, and biologically (2). At the same time, the other two layers depend on inducible responses. Defects in the barrier can give rise to abnormal responses in subsequent layers, resulting in inflammatory skin diseases (1). We have previously proposed a concept that interactions in “a loop” among epithelial–immune microenvironment (EIME)-specific factors—microbiota, barrier, epidermis, immune cells, and peripheral nerve endings—are observed in chronic inflammatory skin diseases such as atopic dermatitis and psoriasis (3) and epidermal keratinocytes play central roles in the initiation and propagation of the inflammatory responses (3, 4). However, a shared molecule in keratinocytes that responds to diverse dangers and stresses and governs the protective machinery of the skin, and induces various types of inflammatory responses remains obscure.

G protein-coupled receptor 15 ligand (GPR15L) is a 9 kDa polypeptide encoded by orphan genes *C10orf99* in humans and *2610528A11Rik* in mice (5, 6). GPR15L is a ubiquitous membrane/secretion protein expressed in epithelial tissues such as gastrointestinal mucosa, cervix, and skin. Its orthologs display a signal peptide, four conserved cysteines forming intramolecular disulfide bridges, and a highly conserved C-terminal domain, which is involved in receptor activation and signaling (5, 6). *2610528A11Rik* deficient mice are fertile and develop normally but exhibit an increased ovalbumin-specific IgG2a response (7). A *C10orf99* peptide has broad antimicrobial activity against different organisms, including *Staphylococcus aureus*, *Aspergillus*, *Mycoplasma*, and lentiviruses (8). In addition, several functions of GPR15L have been suggested in the context of molecular interaction: the binding of *C10orf99* peptides with sushi containing domain-2 (SUSD2) induces the growth inhibition of intestinal epithelial cancer cell lines (9), involvement of *C10orf99* in the insulin growth factor-like (IGFL)–Akt pathway (10), and a chemoattractant activity as the ligand of GPR15, an orphan G protein-coupled receptor (5, 6). Of note, gene expression levels of *C10orf99/2610528A11Rik* are highly increased in the lesional skin of human psoriasis (10–14) and murine psoriasis models (15), as well as in atopic dermatitis (16–19) and its murine models (19). However, details of the mechanical involvement of GPR15L in the barrier and inflammatory responses of the skin remain elusive.

GPR15 was originally identified as a G protein-coupled receptor (GPCR) family member (20). It was identified as a receptor for the infection of simian and human immunodeficiency viruses (21). GPR15 is expressed by memory B cells, plasmablasts, memory/effector and regulatory T cell subsets, and directs their homing to the colon to regulate colitis (22–25). In particular, GPR15 mediates dendritic epidermal T-cell recruitment (5, 6, 26). Specifically, dendritic epidermal T-cell precursors from GPR15-deficient mice failed to migrate in response to GPR15L (6). However, the role of GPR15L–GPR15 interaction in skin inflammation remains controversial. Transgenic overexpression of GPR15L seems to confer significant protection in the murine imiquimod-induced psoriatic dermatitis model (5), which is blunted by local depletion of GPR15L (27). Nevertheless, GPR15 deficiency does not alter the course of disease in an imiquimod-induced or IL-23-induced psoriatic dermatitis model (28). These results suggested more intrinsic functions of GPR15L beyond its antimicrobial activity or GPR15-mediated chemotactic activity in the skin.

Here we report that GPR15L plays a primary role in the innate response of keratinocytes and barrier formation in the skin. GPR15L expression is promptly induced during keratinocyte differentiation while it was impaired by GPR15L treatment *in vitro*. In addition, GPR15L depletion leads to defective lipopolysaccharide (LPS)-induced transcriptional response of keratinocytes *in vitro* and blunted skin inflammation by imiquimod treatment *in vivo*. Furthermore, a drastic increase in GPR15L expression levels is found in a wide variety of skin inflammation regardless of the type of immune response, suggesting a fundamental role for GPR15L in the immunological and structural barrier of the epidermis.

## Materials and Methods

### Animals

Eight- to 12-week-old female mice were used in this study. All animals used were of the C57BL/6 genetic background. *2610528A11Rik*-/- mice have been previously generated in a mouse knockout library for secreted and transmembrane proteins (7). Heterozygous littermates were used as wild-type controls unless otherwise indicated.

### Skin Inflammation Models

For DNFB-induced contact hypersensitivity, mice were sensitized to shaved abdominal skin with 25 μL 0.5% (w/v) DNFB in acetone/olive oil (4:1). Five days after sensitization, the ears were challenged with an application of 20 μL 0.3% DNFB. For the papain occlusive dressing technique, 100 μg of papain (Calbiochem, San Diego, CA, USA) diluted in 10 μL PBS was applied to the shaved and tape-stripped dorsal skin and fixed. Each mouse had a total of three 4-day exposures to the patch, separated by 3-day intervals. Mice were euthanized at the end of the third cycle of sensitization. For the imiquimod-induced psoriasis model, a daily dose of 10 mg of imiquimod-containing cream, Aldara (Beselna Cream 5%, Mochida Pharmaceuticals, Tokyo, Japan), was applied to the mouse ear for 2–6 consecutive days. For irritant contact dermatitis, mice were anesthetized with diethyl ether, and 20 μL of 1% (v/v) croton oil in acetone was applied to the ear skin. All animals were maintained under specific pathogen-free conditions at the Institute of Laboratory Animals at Kyoto University Graduate School of Medicine. The sample sizes were determined based on previous studies (4, 29). The sample size reflects the number of independent biological replicates for each experiment and is provided in the figure legend.

### Human Specimens

We obtained biopsy specimens of skin lesions from patients with atopic dermatitis or psoriasis at Kyoto University Hospital.

### Histological Analyses

Skin tissues were fixed in 10% formalin in PBS and then embedded in paraffin. Sections of 5 μm thickness were prepared and stained with hematoxylin and eosin (H&E). For immunohistochemistry, formalin-fixed, paraffin-embedded tissue samples were processed for immunohistochemistry using the following primary antibodies: anti-C10orf99 antibody (1:500, ab151109, Abcam, Cambridge, UK), anti-filaggrin antibody (1:250, sc-66192, Santa Cruz Biotechnology Inc., Dallas, TX, USA), and anti-loricrin antibody (1:1,000, RPB-145P, Covance, Princeton, NJ, USA). Sections were deparaffinized and processed for antigen retrieval by incubating in 10 mM sodium citrate buffer (pH 6.0) at 95–100°C for 10 min. Tissue samples were incubated overnight with primary antibodies at 4˚C. The sections were incubated with the biotinylated secondary antibody and with streptavidin-HRP (VECTASTAIN^®^ Elite^®^ ABC Kit Peroxidase (HRP) PK-6100, Vector Laboratories, Burlingame, CA), and developed with DAB reagents (Vector^®^ DAB, Vector Laboratories), following the manufacturer’s instructions.

### Quantitative PCR

Total RNA was isolated using TRIzol (Invitrogen, Waltham, MA, USA) or RNeasy kits (Qiagen, Hilden, Germany) according to the manufacturer’s protocol. Complementary DNA was reverse-transcribed using the Prime Script RT reagent kit (Takara Bio, Kusatsu, Japan). Quantitative RT-PCR was performed as previously described (4). All the primers were obtained from Greiner Bio-One Co., Tokyo, Japan. Primer sequences are listed in **Table S1** in the **Supplementary Material**.

### Cell Culture

Mouse primary keratinocytes were isolated from newborn mice, cultured in a low-calcium medium (0.05 mM Ca^2+^), and induced to differentiate by raising calcium to 1.3 mM, as described previously (30). For cell-stimulation experiments, cells were serum-starved for 24 h before stimulation. The culture was maintained at 32°C in a humidified chamber containing 5% CO_2_. NHEKs (Kurabo, Osaka, Japan) were inoculated at a concentration of 2,500 cells/cm^2^ in culture medium, HuMedia-KG2 (Kurabo) supplemented with insulin (10 μg/mL), human epidermal growth factor (0.1 ng/mL), hydrocortisone (0.5 μg/mL), bovine pituitary extract (0.4% v/v), gentamicin (50 μg/mL), and amphotericin B (50 ng/mL). The culture was maintained at 37°C in a humidified chamber containing 5% CO_2_. For the 3D-cultured human epidermis, we used the LabCyte EPI-MODEL 24, 6-day culture kit, as a model for the developing human epidermis (Japan Tissue Engineering Co., Ltd., Gamagori, Japan).

### Peptides

GPR15L synthetic peptides (mouse, 54 AA; human full-length, 57 AA; and human ∆C, 47 AA) were obtained from Zhejiang Ontores Biotechnologies Co., Ltd., Hangzhou, Zhejiang Province, China. The peptides were dissolved in PBS at the indicated concentrations. For animal treatment, peptide solution or PBS were subcutaneously injected into both ears of each mouse using a 30-gauge needle every other day for ten days.

### Transfection Analysis

The human *C10orf99* expression vector was generated by inserting a *C10orf99* open reading frame (NM_207373) into the multiple cloning sites of pIRES-EGFP (Clontech Laboratories, Mountain View, CA, USA). According to the manufacturer’s instructions, the human *C10orf99* expression vector (or the mock vector) was transfected using Lipofectamine 3000 (Thermo Fisher Scientific, Waltham, MA). The cells were harvested for quantitative RT-PCR analyses before and after 2-day culture.

### RNA-Seq Library Preparation

Total RNA was extracted from primary cultured normal human epidermal keratinocytes (NHEK) following the double extraction protocol: RNA isolation by acid guanidinium thiocyanate-phenol-chloroform extraction (TRIzol, Thermo Fisher Scientific, Waltham, MA, USA) followed by a Qiagen RNeasy Micro clean-up procedure (Qiagen, Hilden, Germany). RNA was analyzed on Agilent Bioanalyser for quality assessment with RNA Integrity Number (RIN) range from 9.6 to 9.8 and median of RIN 9.8. cDNA libraries were prepared using 2 ng of total RNA using the SMARTSeq v2 protocol (31) with the following modifications: 1. Addition of 20 µM TSO; 2. Use of 200 pg cDNA with 1/5 reaction of Illumina Nextera XT kit (Illumina, San Diego, CA, USA). The length distribution of the cDNA libraries was monitored using a DNA High Sensitivity Reagent Kit on the Perkin Elmer Labchip (Perkin Elmer, Waltham, MA, USA). All samples were subjected to an indexed paired-end sequencing run of 2x151 cycles on an Illumina HiSeq 4000 system (Illumina) (31 samples/lane).

### Transcriptome Analyses

The 2 × 151 bp paired-end reads from Illumina sequencing were quality checked using fastqc (http://www.bioinformatics.bbsrc.ac.uk/projects/fastqc) and after quality checks mapped to the GRCh38 human genome assembly using the STAR alignment tool (32). Reads mapped to genes were counted in each sample using featureCounts (33), where the reference gene annotations were obtained from GENCODE v26 (34). Gene counts were loaded into the R/Bioconductor package edgeR (35) for differential gene expression analysis. Gene counts were normalized across samples using the trimmed mean of M-values (TMM) method. Genes with expression lower than one count per million in all samples were excluded from the analysis. A negative binomial generalized linear model was fitted to the data. The estimateDisp function was used to estimate the common, trended, and tagwise dispersion terms. The glmFit and glmLRT functions were used to fit a negative binomial generalized log-linear model to the read counts for each gene and conduct a genewise statistical test of the difference in mean expression between sample groups. Differentially expressed genes were selected based on a false discovery rate (Benjamini-Hochberg multiple testing corrected *p*-value) of < 0.05. RNA-seq data were deposited in the Gene Expression Omnibus (GEO) database; accession number GSE189751.

Gene ontology analysis was performed based on the database for annotation, visualization, and integrated discovery (DAVID) (36) and Enrichr (37).

Parametric enrichment analysis based on the KEGG functional hierarchy (38) was performed using the GAGE algorithm (39). Briefly, we compared the average gene expression of the target genes of a TF against that of the whole gene. A weighted t-test procedure was used in the parametric gene set enrichment analysis to weigh the target genes with high-frequency binding. The binding frequency of TFs to their target genes was assessed based on a number of high-throughput chromatin immunoprecipitation (ChIP) experiments obtained from the Gene Expression Omnibus database (GEO, www.ncbi.nlm.nih.gov/geo/). A weighted t-statistic was used as the enrichment score. The p values calculated by GAGE were then corrected for multiple testing using the Benjamini-Hochberg procedure. FuncTree was used to visualize enrichment results (40). Transcription factor enrichment analysis based on numerous ChIP-seq data was performed as previously described (41).

### Statistical Analysis

Unless otherwise indicated, data are presented as the mean ± standard deviation. A two-tailed Student’s t*-*test or an analysis of covariance (ANCOVA) was performed to assess statistical significance. Statistical significance was set at *P* < 0.05 and is indicated in the figures.

## Results

### Lesional Skin From Several Immune Types of Dermatitis Commonly Expresses GPR15L in Mice and Humans

The results of our previous transcriptomic profiling by microarrays on biopsy specimens from four animal models of atopic dermatitis (NC/Nga, flaky tail, *Flg*-mutated, and ovalbumin-challenged mice) revealed that *2610528A11Rik* encoding GPR15L is one of four genes, whose expression levels were increased in all the models (19).

**Figure 1:**
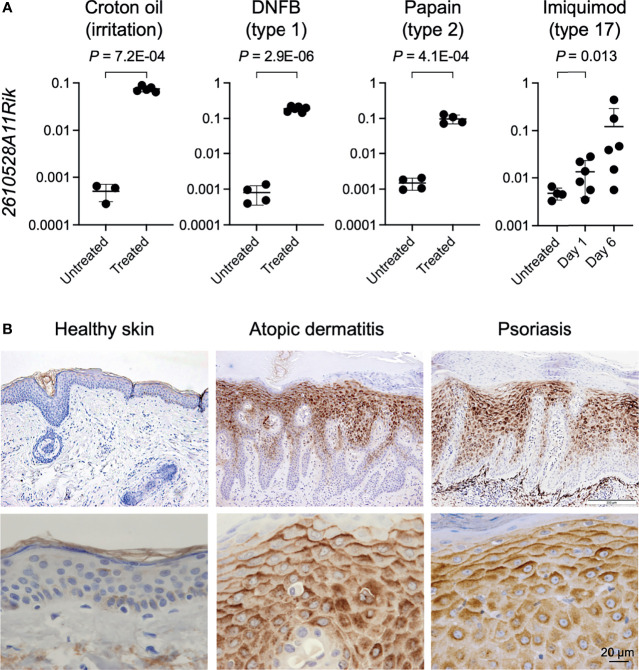
GPR15L expression in the skin with several types of inflammation in mice and humans. **(A)** Quantitative RT-PCR analysis of GPR15L mRNA levels in the lesional skin of mice with helper T lymphocyte-independent irritant dermatitis and with three immune types of skin inflammation. Results were normalized to glyceraldehyde-3-phosphate dehydrogenase (*Gapdh*) expression (error bars, SD; n ≥ 5 per group). **(B)** GPR15L protein expression in healthy human skin and lesional skin from patients with atopic dermatitis or psoriasis. Representative results from the three or more cases are shown. Upper panels, low power (a scale bar, 200 µm); Lower panels, high power (a scale bar, 20 µm).

We next wonder whether the induction of GPR15L expression in the lesional skin is widely shared in mice with several inflammatory skin conditions with different immune types. We analyzed the gene expression levels of *2610528A11Rik* in the lesional skin of mice with croton oil-induced irritant dermatitis (a model for T cell-independent innate response) (42), DNFB-induced contact hypersensitivity (a model for allergic contact dermatitis; type 1 immunity) (43), papain-induced dermatitis (a model for atopic dermatitis; type 2 immunity) (44), and imiquimod-induced dermatitis (a model for psoriasis; type 17 immunity) (45). We found that gene expression levels of *2610528A11Rik* were more than 100 times higher in lesional skin with croton oil-induced irritant contact dermatitis than in healthy skin (**Figure 1A**). The higher expression levels of GPR15L in the lesional skin were common to all the other dermatitis models (**Figure 1A**).

Next, we evaluated the protein expression levels of GPR15L in the lesional skin of humans with representative inflammatory skin diseases, including atopic dermatitis and psoriasis, since the increase in gene expression levels of GPR15L in the lesional skin has been demonstrated in multiple transcriptomic studies in both diseases (10–14, 16–19). As expected, immunohistochemistry revealed much higher expression levels of GPR15L protein in the lesional skin of patients with atopic dermatitis or psoriasis than in healthy skin (**Figure 1B**). Specifically, the induction of GPR15L is mainly localized in the epidermis. In addition, its expression levels were higher in the suprabasal cells (especially in the upper layer) than in the basal cells of the epidermis and higher on the upper side than on the lower side of each epidermal keratinocyte (**Figure 1B**). These patterns were similar to the expression of human β-defensin 2, a microbial peptide remarkably induced in the epidermis of patients with psoriasis (46) though the intracellular expression patterns of GPR15L in the epidermis were unclear in a previous study (27). Regardless of immune cell infiltration, there was incontinence of GPR15L into the extracellular spaces of the papillary dermis of the lesional skin from some patients with psoriasis (**Figure S1B**).

These results suggest that GPR15L is a highly inducible molecule in epidermal keratinocytes in response to skin inflammation, regardless of the immune type of inflammation.

### High Calcium-Induced Differentiation Triggers GPR15L Expression in Mouse Primary Keratinocytes

**Figure 2:**
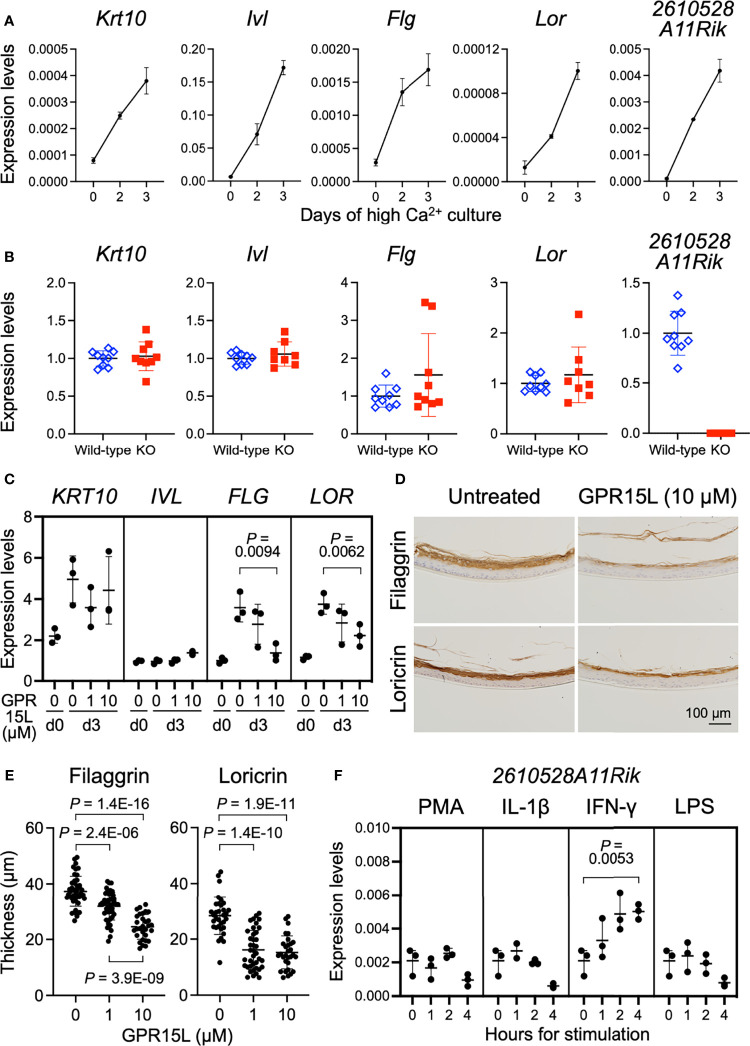
GPR15L expression in keratinocytes and GPR15L-mediated control for epidermal differentiation. **(A)** Quantitative RT-PCR analysis of mRNA levels of keratinocyte differentiation markers and GPR15L in mouse primary cultured keratinocytes during Ca^2+^-induced differentiation. Results were normalized to *Gapdh* expression (error bars, SD; n = 3 per group). **(B)** Quantitative RT-PCR analysis of mRNA levels of keratinocyte differentiation markers in primary cultured keratinocytes from wild-type or GPR15L-null mice on day three of Ca^2+^-induced differentiation. Results were normalized to *Gapdh* expression and indicated as expression levels relative to those of the wild-type (error bars, SD; n ≥8 per group). **(C)** Quantitative RT-PCR analysis of mRNA levels of keratinocyte differentiation markers in 3D-cultured human epidermis treated with GPR15L during development *in vitro*. Results were normalized to *GAPDH* expression (error bars, SD; n = 3 per group). **(D)** Protein expression levels of keratinocyte differentiation markers in 3D-cultured human epidermis treated with or without 10 µM GPR15L during development *in vitro*. Representative results from triplicates are shown (a scale bar, 100 µm). **(E)** Thickness of the filaggrin-positive and loricrin-positive layers in the 3D-cultured human epidermis treated with or without GPR15L during development *in vitro*. Thickness of filaggrin or loricrin-positive layers of three sections from each of the triplicates were measured at three or more sites (error bars, SD; n ≥ 30 per group). **(F)** Quantitative RT-PCR analysis of GPR15L mRNA levels in mouse primary cultured keratinocytes with indicated stimulations. Results were normalized to *Gapdh* expression (error bars, SD; n = 3 per group).

The mechanism of GPR15L gene expression induction has not yet been fully elucidated. In newborn mice, GPR15L was mainly expressed in the epidermis (**Figure S1A**). Therefore, we first evaluated the nature of GPR15L gene expression in mouse keratinocytes during calcium-induced differentiation *in vitro*. Switching experimentally from low to high calcium induces phosphoinositide-dependent kinase 1 (PDK1)-dependent cell differentiation in keratinocytes (30). Quantitative RT-PCR showed that GPR15L gene expression as well as gene expression of *Krt10* (encoding keratin 10), a suprabasal cell marker, and *Ivl* (involucrin), *Flg* (filaggrin), and *Lor* (loricrin), late differentiation markers, was induced by high calcium levels in primary cultured mouse keratinocytes (**Figure 2A**). These results suggest that GPR15L expression is triggered by keratinocyte differentiation during the basal-to-suprabasal switch or a late differentiation phase mediating barrier formation in the epidermis.

### GPR15L Treatment *In Vitro* Attenuates Late Keratinocyte Differentiation

Gene expression levels of keratinocyte differentiation markers in newborn epidermis were comparable between wild-type and *2610528A11Rik* deficient mice (**Figure S2A**). To investigate the relationship between GPR15L expression and keratinocyte differentiation, we evaluated the calcium-induced differentiation of GPR15L-deficient keratinocytes. On day three of the high-calcium culture conditions, the gene expression levels of *Krt10*, *Ivl*, *Flg*, and *Lor* were comparable between GPR15L-deficient keratinocytes and wild-type keratinocytes (**Figure 2B**), although only a few specimens revealed higher levels of *Flg*, and *Lor*, late differentiation markers for keratinocytes, in GPR15L-deficient keratinocytes.

To further analyze the relationship between GPR15L and barrier formation of the epidermis, we treated 3D-cultured normal human epidermal keratinocytes with GPR15L synthetic peptides during their stratification *in vitro*. GPR15L treatment downregulated the gene expression levels of filaggrin and loricrin in a dose-dependent manner (**Figure 2C**). Consistently, immunohistochemistry of the 3D-cultured epidermis showed that protein expression levels of filaggrin and loricrin were lower in the GPR15L-treated group than in the non-treated group (**Figure 2D**). In addition, thickness of the filaggrin-positive layers in the 3D-culturted epidermis were less in the GPR15L-treated group in a dose-dependent manner (**Figure 2E**). Thickness of the loricrin-positive layers were also less in the GPR15L-treated group in both 1 µM and 10 µM concentrations than in the non-treated group (**Figure 2E**).

These results suggest that GPR15L negatively regulates the late differentiation of keratinocytes and barrier formation of the epidermis. In contrast, defective GPR15L does not affect normal keratinocyte differentiation.

Next, we tested several stimulations or cellular stresses to induce GPR15L gene expression in keratinocytes. We stimulated mouse primary cultured keratinocytes with NF-κB activators, phorbol 12-myristate 13-acetate (PMA), interleukin (IL)-1β, interferon (IFN)-γ, or lipopolysaccharide (LPS) because the *2610528A11Rik* gene has several κB sites in the promoter region and the first intron. All these stimuli induced IL-1β transcription in mouse primary cultured keratinocytes by 4 hours (**Figure S2B**). We found that the up-regulation of GPR15L gene expression in mouse keratinocytes was detectable in 1 hour and enhanced by 4 hours after stimulation with IFN-γ *in vitro* (**Figure 2F**). In contrast, the other stimuli or stress conditions failed to induce GPR15L expression (**Figure 2F**). These results suggest that GPR15L expression in keratinocytes is induced during cell-intrinsic differentiation and is augmented by a certain external inflammatory mediator, such as IFN-γ.

### GPR15L Gene Transfection Induces Inflammatory Gene Expression and Reduces Barrier Gene Expression in Normal Human Epidermal Keratinocytes

**Figure 3:**
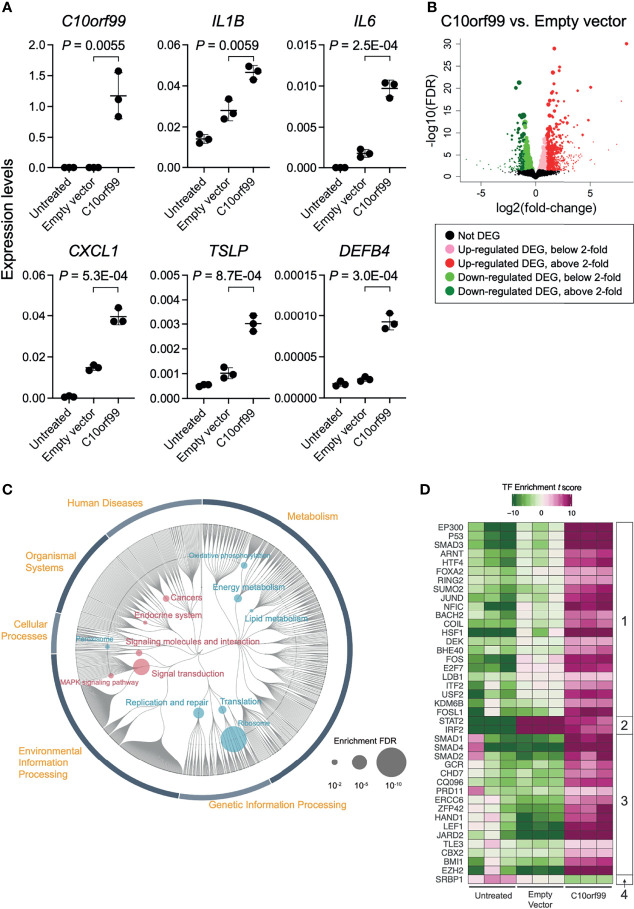
Gene expression profiles in normal human epidermal keratinocytes (NHEKs) transfected with *C10orf99*. **(A)** Quantitative RT-PCR analysis of mRNA levels in untreated NHEKs transfected with empty vector or with *C10orf99*. The results were normalized to *GAPDH* expression levels (error bars, SD; n = 3). **(B)** Volcano plots of the relative differences in gene expression levels using the differentially expressed genes (DEGs) in NHEKs transfected with empty vector or *C10orf99* and analyzed by RNA-seq analysis. Each point represents a unique gene, and the size of a point is proportional to the average absolute expression level of the gene (log2 CPM) over all samples. **(C)** Parametric enrichment analysis of the DEGs. The analysis was based on the KEGG functional hierarchy. **(D)** Transcription factor (TF) enrichment analysis of DEGs. The analysis was based on ChIP-seq data. The TFs were divided into four groups based on their changes in the enrichment *t* scores.

The inducible responses of keratinocytes against external dangers and stresses have been demonstrated to play a critical role in triggering and propagating different types of skin inflammation (4, 47). To evaluate the role of GPR15L in the proinflammatory response of keratinocytes, we transfected NHEKs with GPR15L-expressing plasmids or empty plasmids. We found that the gene expression levels of representative proinflammatory mediators, such as IL-1β, IL-6, chemokine C-X-C motif ligand 1 (CXCL1), thymic stromal lymphopoietin (TSLP), and β-defensin 4, from keratinocytes after the overnight transfection were higher in GPR15L-expressing NHEKs than in empty plasmid-transfected NHEKs (**Figure 3A**).

To further characterize the GPR15L-induced transcriptional response of keratinocytes, we performed RNA-Seq analyses on keratinocytes. The quality of transcriptomes from untreated NHEKs, empty vector-transfected NHEKs, or *C10orf99*-transfected NHEKs (n = 3) was validated by sample clustering analyses (**Figure S3**). We detected 2,186-differentially expressed genes (DEGs) between the *C10orf99*-transfected NHEKs and empty plasmid-transfected NHEKs (**Figure 3B** and **Table S2**). The results of database for annotation, visualization, and integrated discovery (DAVID) and Enrichr gene ontology analyses suggested the highest relation of C10orf99 to “lipid/cholesterol biosynthesis, followed by “cadherin/cell adhesion,” and “mitochondrion” (**Table S3**).

The up-regulated DEGs in the GPR15L-expressing NHEKs included a set of genes related to skin inflammation: *CSF1* (encoding M-CSF, macrophage colony-stimulating factor), *IL24* (IL-24), *SOCS3*, *IL6* (IL-6), *MAP3K11, CSF2* (GM-CSF, granulocyte-macrophage colony-stimulating factor), *IL20* (IL-20)*, RELB, IL7R* (a receptor for IL-7) and *TSLP* (thymic stromal lymphopoietin), *PTGER4* (prostaglandin E2 receptor EP4), *CIITA*, and *SEMA3E* (**Table 1**). In addition, genes encoding mitochondrial proteins, such as *MARC1*, *PREP*, and *COQ2*, were highly up-regulated (**Table 1**). Of note, some genes whose known functions have not been investigated in keratinocytes are listed in **Table 1**; for example, *MAFF*, a transcriptional cofactor for keratinocyte differentiation (48), was recently reported as a central regulator of atherosclerosis connecting inflammation and cholesterol metabolism (49). *LINC00702*, a long-noncoding RNA, regulates *PTEN via* miR-510, suppresses proliferation and invasion in non-small cell lung cancer (50), and activates the Wnt/β-catenin pathway during meningioma progression (51).

**Table 1:** Thirty representative DEGs up-regulated in the GPR15L-transfected NHEKs.

Symbol	Fold*	logCPM	LR	P-Value	FDR	Protein	Category**
***C10orf99***	**326.97**	6.66	588.23	6.09E-130	9.83E-126	chromosome 10 open reading frame 99	–
***CTGF***	**32.95**	6.05	103.23	2.99E-24	6.03E-21	connective tissue growth factor	KC differentiation
***DUSP1***	**28.95**	4.95	66.37	3.74E-16	1.89E-13	dual specificity phosphatase 1	Inflammation
***SDF4***	**20.12**	4.77	49.91	1.61E-12	3.42E-10	stromal cell derived factor 4	KC differentiation
***INA***	**15.79**	2.03	34.66	3.92E-09	3.42E-07	internexin neuronal intermediate filament protein alpha	Others
***MAFF***	**14.77**	4.32	99.02	2.50E-23	4.03E-20	MAF bZIP transcription factor F	Others
***MARC1***	**11.77**	2.00	58.63	1.91E-14	6.28E-12	mitochondrial amidoxime reducing component 1	Mitochondria
***LINC00702***	**8.74**	2.30	25.52	4.38E-07	2.18E-05	(long intergenic non-protein coding RNA 702)	Others
***CSF1***	**7.39**	2.51	62.13	3.21E-15	1.20E-12	colony stimulating factor 1	Inflammation
***PREP***	**6.56**	6.18	69.31	8.40E-17	4.52E-14	prolyl endopeptidase	Mitochondria
***COQ2***	**6.10**	4.35	20.57	5.74E-06	1.83E-04	coenzyme Q2, polyprenyltransferase	Mitochondria
***BTG1***	**5.95**	5.18	64.32	1.06E-15	4.87E-13	BTG anti-proliferation factor 1	Others
***IL24***	**4.61**	5.59	126.04	3.02E-29	1.62E-25	interleukin 24	Inflammation
***MMP3***	**4.50**	2.67	56.37	6.02E-14	1.77E-11	matrix metallopeptidase 3	KC differentiation
***CAPN8***	**4.34**	2.48	49.60	1.89E-12	3.96E-10	calpain 8	Inflammation
***TRAF4***	**4.24**	4.90	87.18	9.90E-21	8.87E-18	TNF receptor associated factor 4	Inflammation
***SOCS3***	**3.18**	2.18	18.18	2.01E-05	5.26E-04	suppressor of cytokine signaling 3	Inflammation
***IL6***	**3.05**	2.08	27.16	1.87E-07	1.02E-05	interleukin 6	Inflammation
***NGF***	**3.04**	2.02	12.49	4.10E-04	6.04E-03	nerve growth factor	Others
***MAP3K11***	**3.00**	3.59	12.42	4.24E-04	6.17E-03	mitogen-activated protein kinase kinase kinase 11	Inflammation
***CSF2***	**2.84**	2.22	21.38	3.77E-06	1.30E-04	colony stimulating factor 2	Inflammation
***IL20***	**2.81**	2.47	26.15	3.16E-07	1.63E-05	interleukin 20	Inflammation
***TXNRD3***	**2.67**	3.24	32.90	9.70E-09	7.83E-07	thioredoxin reductase 3	Inflammation
***RELB***	**2.60**	2.45	18.18	2.01E-05	5.26E-04	RELB proto-oncogene, NF-kB subunit	Inflammation
***IL7R***	**2.50**	3.54	20.23	6.87E-06	2.12E-04	interleukin 7 receptor	Inflammation
***MAP2K5***	**2.26**	3.81	18.06	2.14E-05	5.49E-04	mitogen-activated protein kinase kinase 5	Inflammation
***PTGER4***	**2.24**	3.84	12.79	3.49E-04	5.32E-03	prostaglandin E receptor 4	Inflammation
***CIITA***	**2.22**	2.13	11.18	8.26E-04	1.05E-02	class II major histocompatibility complex transactivator	Inflammation
***SEMA3E***	**2.15**	3.03	13.26	2.70E-04	4.40E-03	semaphorin 3E	Inflammation
***JUN***	**2.14**	7.61	28.73	8.34E-08	4.98E-06	proto-oncogene c-Jun	Inflammation

*Shading with orange ≥ 10, light orange ≥ 3. **Colored according to the category.

The down-regulated DEGs in the GPR15L-expressing NHEKs included a set of genes related to keratinocyte differentiation and cell adhesion: *CASP14* (coding caspase 14), *LY6D*, *DSG1* (desmoglein 1), *IVL* (involucrin), and *DSC1* (desmocollin 1) (**Table 2**). A series of psoriasis-related genes, such as *S100A8*, *S100A9*, *KRT6A* (keratin 6A), *KRT6B*, *KRT6C*, *KRT16*, and *MMP9* (metalloproteinase 9), were also down-regulated (**Table 2**). In particular, several genes related to barrier formation of the stratum corneum, such as *TGM1* (transglutaminase 1), *SERPINB3* (serpin family B member 3), and *SPINK5* (LEKTI, lympho-epithelial Kazal-type-related inhibitor), were down-regulated (**Table 2**). In addition, a set of genes related to cholesterol and lipid metabolism, such as *HSD3B7*, *PLA2G4E*, *SULT2B1*, and *ALOX15B*, were down-regulated (**Table 2**). Of note, a set of genes related to antiviral response, such as *RAB7B* (Ras-related protein Rab-7b), *IFIT3* (interferon-induced protein with tetratricopeptide repeats 3), *IFIT2*, and *IFIT1*, were included in the down-regulated DEGs (**Table 2**).

**Table 2:** Thirty representative DEGs down-regulated in the GPR15L-transfected NHEKs.

Symbol	Fold*	logCPM	LR	PValue	FDR	Protein	Category**
***CASP14***	**0.22**	4.20	45.11	1.87E-11	3.11E-09	caspase 14	KC differentiation
***RAB7B***	**0.22**	4.60	49.23	2.28E-12	4.62E-10	RAB7B, member RAS oncogene family	Inflammation
***CDHR1***	**0.23**	2.25	23.19	1.47E-06	6.04E-05	cadherin related family member 1	KC differentiation
***S100A8***	**0.27**	3.11	55.50	9.34E-14	2.47E-11	S100 calcium binding protein A8	Inflammation
***LY6D***	**0.27**	3.80	32.86	9.91E-09	7.96E-07	lymphocyte antigen 6 family member D	KC differentiation
***IFIT3***	**0.28**	8.40	102.78	3.75E-24	6.72E-21	interferon induced protein with tetratricopeptide repeats 3	Inflammation
***S100A9***	**0.32**	4.64	63.63	1.50E-15	6.55E-13	S100 calcium binding protein A9	Inflammation
***KRT6B***	**0.35**	10.66	108.92	1.69E-25	4.54E-22	keratin 6B	KC differentiation
***HSD3B7***	**0.35**	2.61	19.35	1.09E-05	3.08E-04	3 beta-hydroxysteroid dehydrogenase type 7	Lipid
***TGM1***	**0.35**	7.07	57.17	3.99E-14	1.24E-11	transglutaminase 1	KC differentiation
***PLA2G4E***	**0.35**	2.31	16.76	4.25E-05	9.90E-04	phospholipase A2 group IVE	Lipid
***TAF9B***	**0.36**	4.62	46.18	1.08E-11	1.87E-09	TATA-box binding protein associated factor 9b	Others
***SPRR1B***	**0.36**	5.99	42.34	7.67E-11	1.10E-08	small proline rich protein 1B	KC differentiation
***SERPINB3***	**0.36**	3.97	33.52	7.07E-09	5.91E-07	serpin family B member 3	KC differentiation
***GJB6***	**0.37**	3.86	36.88	1.25E-09	1.37E-07	gap junction protein beta 6	KC differentiation
***GJB2***	**0.37**	8.41	72.54	1.64E-17	1.20E-14	gap junction protein beta 2	KC differentiation
***MMP9***	**0.38**	3.22	23.99	9.68E-07	4.28E-05	matrix metallopeptidase 9	KC differentiation
***IFIT2***	**0.39**	7.64	27.60	1.49E-07	8.21E-06	interferon induced protein with tetratricopeptide repeats 2	Inflammation
***SULT2B1***	**0.39**	4.22	20.39	6.31E-06	1.97E-04	sulfotransferase family 2B member 1	Lipid
***ALOX15B***	**0.41**	5.78	47.39	5.81E-12	1.07E-09	arachidonate 15-lipoxygenase, type B	Lipid
***DSG1***	**0.41**	3.11	18.15	2.04E-05	5.30E-04	desmoglein 1	KC differentiation
***A2ML1***	**0.42**	6.29	48.60	3.14E-12	6.02E-10	alpha-2-macroglobulin like 1	KC differentiation
***IVL***	**0.42**	4.26	25.99	3.42E-07	1.75E-05	involucrin	KC differentiation
***KRT6C***	**0.42**	9.59	69.90	6.25E-17	3.47E-14	keratin 6C	KC differentiation
***IFIT1***	**0.43**	8.59	31.23	2.29E-08	1.64E-06	interferon induced protein with tetratricopeptide repeats 1	Inflammation
***KRT16***	**0.43**	9.75	50.04	1.51E-12	3.25E-10	keratin 16	KC differentiation
***KRT6A***	**0.46**	13.58	72.19	1.96E-17	1.35E-14	keratin 6A	KC differentiation
***SPINK5***	**0.49**	2.76	12.17	4.87E-04	6.86E-03	serine peptidase inhibitor, Kazal type 5	KC differentiation
***KRT15***	**0.50**	6.94	29.06	7.00E-08	4.26E-06	keratin 15	KC differentiation
***DSC1***	**0.50**	2.28	8.30	3.97E-03	3.39E-02	desmocollin 1	KC differentiation

*Shading with blue ≤ 0.3, light blue ≤ 0.4. ** Colored according to the category.

These results suggest that GPR15L induces inflammatory gene expression and reduces barrier gene expression in epidermal keratinocytes.

### Pathway Analysis: GPR15L Induces Signal Transduction and Reduces Energy and Lipid Metabolism in Keratinocytes

To characterize the impact of GPR15L in keratinocytes more comprehensively, we performed Kyoto Encyclopedia of Genes and Genomes (KEGG) pathway analysis and transcription factor enrichment analysis of the RNA-Seq data. We compared the results of *C10orf99*-transfected NHEKs to those of the empty vector-transfected NHEKs unless otherwise indicated because lipofection induced an inflammatory response, which was not found in untreated NHEKs, in both groups.

The results of KEGG pathway analysis revealed that genes related to “Signal transduction” and “Signaling molecules and interaction,” especially “Mitogen-activated protein kinase (MAPK) signaling pathway,” were up-regulated by the expression of GPR15L in NHEKs (**Figure 3C**, and **Table S4**). On the other hand, down-regulation of genes related to “Energy metabolism,” “Lipid metabolism,” and “Translation” was considered to be a unique change in GPR15L-transfected NHEKs (**Figure 3C**, and **Table S4**). At the same time, the relation to ribosome might not correspond to the gene function in this analysis.

### TF Enrichment Analysis: GPR15L Enhances Stress-Inducible Transcription

The results of transcription factor (TF) enrichment analysis indicated average changes in the expression levels of their target genes. We detected 40 TFs that showed significant changes in the TF enrichment *t* scores in untreated and *C10orf99*-transfected NHEKs, and empty vector vs. *C10orf99*-transfected NHEKs (**Figure 3D**).

They were divided into four groups based on their changes in the TF enrichment *t* scores among each condition: *Group 1*, down in untreated, up in empty vector, and more up in C10orf99; *Group 2*, down in untreated, up in empty vector, and less up in C10orf99; *Group 3*, comparable or less in untreated and empty vector, and up in C10orf99; and *Group 4*, up in untreated and down in C10orf99 (**Figure 3D**).

In *Group 1*, there were a set of ubiquitous stress-inducible TFs, such as *EP300* (coding p300), *SMAD3*, *ARNT* (aryl hydrocarbon receptor nuclear translocator), *JUND*, *FOS*, and *HSF1* (heat shock transcription factor 1) (**Figure 3D**). TF p300 is an acetyl-transferase with chromatin binding and subsequent histone acetylation as key functions for the transcriptional activation of stress-inducible genes (52). In addition, *P53*, a tumor suppressor that induces growth arrest or apoptosis *via* chromatin remodeling, was also included in this group. These results indicate that genes that respond to these TFs are strictly regulated in untreated keratinocytes but are released from this regulation under stress conditions by lipofection and their expression is further enhanced by GPR15L expression.

In *Group 2*, *STAT2* and *IRF2* showed a strong increase in the empty vector group compared to the untreated group, despite a weak increase in the *C10orf99*-transfected group (**Figure 3D**). These results indicate that transcription of STAT2- and IRF2-target genes is from a group of lipofection-induced stress responses, which GPR15L negatively regulates. Both STAT2 and IRF2 are negative regulators of type I IFN signaling (53, 54). These results suggest that GPR15L inhibits the transcription of the stress-induced group 2-target genes by direct inhibition of type I IFN signaling or indirect inhibition by enhancing STAT2- or IRF2-mediated transcriptional regulation.

In *Group 3*, *SMAD1*, *SMAD2*, *SMAD4*, *JARD2*, and *EZH2* indicate the GPR15L standalone role for their response genes regardless of lipofection-mediated changes (**Figure 3D**). In particular, *JARD2* (JARID2, jumonji and AT-rich interaction domain containing 2) and *EZH2* increased in the enrichment score in the *C10orf99*-transfected group despite decreasing the enrichment score in the empty vector group (**Figure 3D**). JARID2 and EZH2 are components of the polycomb complex 2 (PRC2), a negative regulator of gene transcription *via* chromatin remodeling and histone modification (55). These results suggest that GPR15L may be involved in SMAD-mediated transcription and PRC2-mediated transcriptional regulation, regardless of the stress conditions.

In *Group 4*, only one gene, *SRBP1* (SREBP1, sterol regulatory element-binding protein 1), was included. SREBP1 is a key transcription factor that regulates the expression of genes involved in cholesterol biosynthesis and lipid homeostasis (56). Therefore, SREBP1 is expected to be a major downstream effector of GPR15L in the regulation of lipid metabolism.

### Inflammatory Skin Response is Impaired by GPR15L Deficiency and Induced by GPR15L Treatment

**Figure 4:**
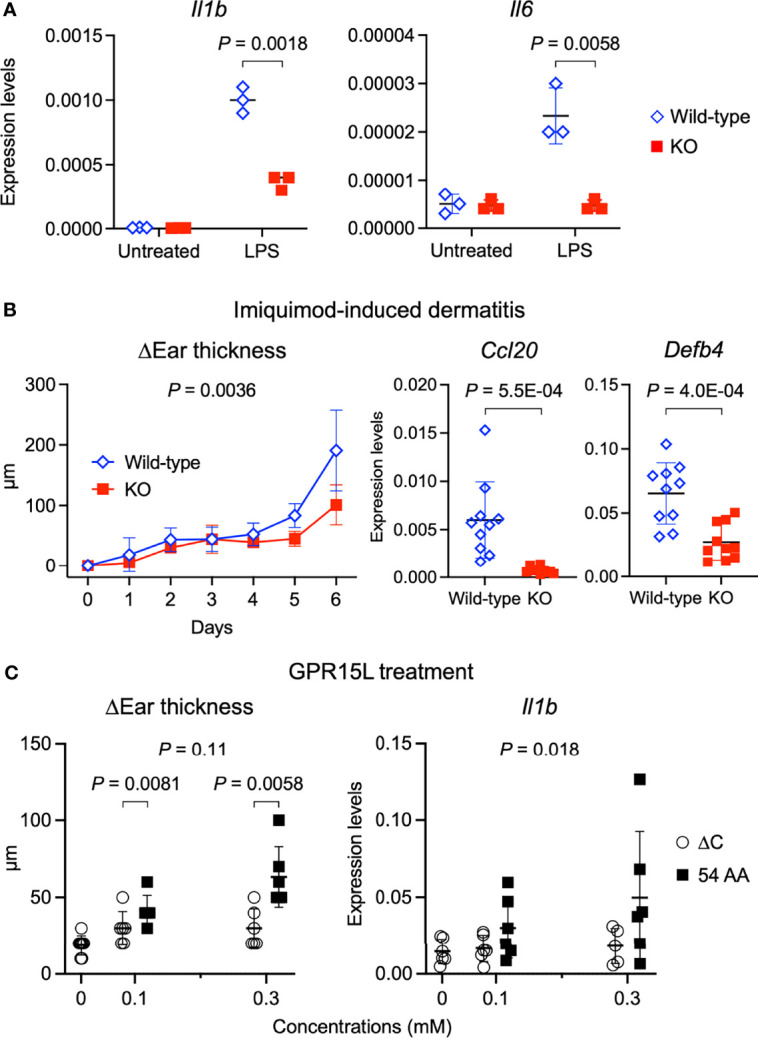
GPR15L deficiency impairs inflammatory responses of keratinocytes and in the skin. **(A)** Quantitative RT-PCR analysis of mRNA levels in primary cultured keratinocytes from GPR15L-deficient (KO) mice or their +/+ littermates (wild-type) with or without LPS stimulation. Results were normalized to *Gapdh* expression (error bars, SD; n = 3). **(B)** Time course of changes in the ear thickness (n ≥ 5 per group) from wild-type or GPR15L-deficient (KO) mice treated daily with or without topical imiquimod for six consecutive days. The thickness was measured daily before treatment. Values are shown as means ± SD. The difference in two curves were evaluated by ANCOVA. *Ccl20* and *Defb4* mRNA levels in the lesional skin from wild-type or GPR15L-deficient (KO) mice treated daily with topical imiquimod for six consecutive days. RT-PCR results were normalized to *Gapdh* expression (error bars, SD; n ≥ 6 per group). We performed RT-PCR experiments with 6-month-old GPR15L deficient mice and wild-type C57BL/6 mice that were co-housed for 1 week before the experiments. The results were reproducible in the two experiments. **(C)** Changes in the ear thickness and *Il1b* mRNA levels analyzed by quantitative RT-PCR (n ≥ 5 per group) from mice intracutaneously treated with GPR15L lacking the C-terminus (∆C) or the full-length (54 AA) every other day for ten days. RT-PCR results were normalized to *Gapdh* expression (error bars, SD; n ≥ 5 per group). The differences in dose-dependent curves between two groups was evaluated by ANCOVA and the differences in each dose between two groups were evaluated by unpaired t-test.

We addressed whether GPR15L has a keratinocyte-intrinsic role in the induction of skin inflammation. We stimulated primary-cultured, wild-type mouse keratinocytes with LPS, which *per se* did not induce the GPR15L expression (**Figure 2F**). Stimulation of mouse primary keratinocytes with LPS triggered the transcription of pro-inflammatory mediators, such as IL-1β and IL-6 (**Figure 4A**). However, the expression levels of *Il1b* encoding IL-1β were lower in the GPR15L-deficient keratinocytes than in the wild-type keratinocytes after LPS stimulation (**Figure 4A**). In addition, the induction of *Il6* transcription was almost completely defective in GPR15L-deficient keratinocytes (**Figure 4A**). These results suggest that GPR15L plays an essential role in the LPS-induced response of keratinocytes.

Next, we tested whether GPR15L has a positive role in the induction of skin inflammation in an animal model of psoriasis because the role of GPR15L in psoriatic skin inflammation remains controversial (27, 28). The increased ear thickness in the psoriatic lesion was milder, and the expression levels of inflammatory genes, *Ccl20* and *Defb4*, in the lesional skin were lower, in the GPR15L-deficient mice than in the wild-type mice (**Figure 4B**). These findings suggest that GPR15L contributes to the development of skin inflammation under a certain pathophysiological condition.

Finally, we tested whether the cutaneous administration of synthetic peptides of GPR15L can induce dermatitis *in vivo*. Subcutaneous injection of GPR15L in mouse ears every other day for ten consecutive days induced a significant increase in ear thickness (**Figure 4C**). However, administration of GPR15L lacking a C-terminal region, which is conserved across species, failed to induce skin inflammation (**Figure 4C**), although their dose-dependent increases in ear thickness were not statistically different. The dose-dependent increase in IL-1β gene expression levels in the GPR15L-treated skin was statistically significant (**Figure 4C**). These results suggest that GPR15L is involved in the proinflammatory response of keratinocytes and the development of inflammatory skin conditions.

## Discussion

We propose a new role for GPR15L in the proinflammatory response and late differentiation of keratinocytes, beyond its previously reported role, such as a chemoattractant (5, 6, 26), and an antimicrobial peptide (8). Our results highlight a keratinocyte-intrinsic role for GPR15L. In NHEK or primary cultured mouse keratinocytes, GPR15L increased the gene expression levels of proinflammatory molecules and reduced the expression levels of barrier molecules. In contrast, GPR15L-deficient keratinocytes showed a defective response to LPS. Previous findings and our results indicate that GPR15L is a highly inducible peptide on a variety of skin inflammatory disorders. These results suggest that GPR15L has new roles in the protective response and barrier formation of the epidermis.

The findings of the new role of GPR15L prompted us to consider whether GPR15L drives these roles in keratinocytes in a GPR15-dependent or a GPR15-independent manner. It has been shown that expression of GPR15 is selective in T lymphocytes (22, 23) and detectable in macrophages, monocytes, and neutrophils (57). In contrast, we could not detect GPR15 gene expression by RT-qRCR and RNA-Seq analyses in NHEK and mouse primary cultured keratinocytes but detected it in skin tissues. We cannot rule out the possible involvement of the GPR15 pathway in keratinocytes in an autocrine manner. However, these findings suggest the GPR15-independent roles for GPR15L in keratinocytes.

Previous studies and our present study proposed possible GPR15-independent roles for GPR15L. It has been demonstrated that antibody-mediated skin inflammation is alleviated in GPR15-deficient mice (58) while imiquimod-induced psoriatic dermatitis is not blunted (28). However, treatment of GPR15L-siRNA reduced the skin inflammation in the imiquimod-induced psoriatic dermatitis model at the treated sites, and the treatment with lentiviral GPR15L overexpression increased the disease severity in this animal model (27). Consistently, we demonstrated that the development of imiquimod-induced psoriatic dermatitis was milder in GPR15L-deficient mice. In addition, the results of TF enrichment analysis in the present study suggested a broader role for GPR15L in keratinocyte transcriptional responses. It is unclear whether all these responses are driven by the known GPR15 pathway reducing cyclic AMP (cAMP) production *via* Gi/o-coupled (59). These findings suggest that GPR15L may contribute to skin inflammation and barrier regulation beyond the GPR15L–GPR15 axis.

Our results suggest that the induction of GPR15L is involved in the impaired barrier during skin inflammation. In contrast, it is not essential for the development and maintenance of steady states. Our *in vitro* study demonstrated the impaired expression of the barrier proteins by GPR15L. Still, we failed to reveal an increase in GPR15L-null keratinocytes. These findings are consistent with the normal skin development in GPR15L-deficient mice (5). However, the extremely lower expression levels of GPR15L in healthy skin compared to inflamed skin suggest that GPR15L expresses and governs its major functions under stressful conditions. Its activity is strictly controlled in healthy states.

The precise mechanism for the induction of GPR15L expression in keratinocytes during skin inflammation remains to be elucidated. We demonstrated that GPR15L is highly inducible during skin inflammation in both mice and humans, regardless of the immune type. Our *in vitro* results suggest that the IFN-γ pathway is a possible upstream inducer of GPR15L. Activation of the IFN-γ pathway triggers the transcription of a series of target genes through activation of the canonical NF-κB pathway (60). Consistently, there are several κB sites in the promoter region and the first intron of *2610528A11Rik*. However, other stimuli that activate the canonical NF-κB pathway, such as LPS, failed to trigger GPR15L transcription in keratinocytes (**Figure 2F**). Other mechanisms or common triggers under stressful conditions may be involved in inducible GPR15L expression at the outermost surface of the body. Our findings also stimulate us to investigate the dynamics of GPR15L expression levels during pathological and protective conditions, such as superficial skin infections and wound healing.

The GPR15L–SREBP1 axis may be critical in the crosstalk between lipid metabolism and inflammation, the regulation of which has been proposed for a long time (61–63). We previously demonstrated that topical activation of cholesterol metabolism *via* the liver X receptor (LXR) alleviates imiquimod-induced psoriatic dermatitis. In contrast, LXR expression levels are decreased in the lesional skin and human psoriasis (64). LXR binds to the SREBP1c promoter and initiates SREBP1c transcription (65). In activated CD4^+^ T cells, the T cell receptor/CD28–mammalian target of rapamycin complex 1 (mTORC1) signaling axis controls fatty acid uptake and biosynthesis through the induction of PPARγ and the activation of SREBP1 (66). In the kidney, SREBP1 regulates transforming growth factor-β (TGF-β) activity *via* a positive feedback loop with the TGF-β–Smad3 pathway in mediating fibrosis (67). Therefore, it would be of great interest to know whether GPR15L addresses the LXR pathway and TGFβ–Smad3 *via* GPR15, unidentified receptors, or intracellularly.

There are a few limitations in the present study. First, our findings *in vivo* are not only explained by the new roles of GPR15L in keratinocytes found in the present study *in vitro*. Specifically, blunted imiquimod-induced dermatitis in GPR15L-deficient mice, or the GPR15L peptide injection-induced dermatitis, could in part be explained by the known role of GPR15L in the GPR15L–GPR15 axis and subsequent recruitment of dendritic epidermal T-cells (5, 6, 26), or in keratinocyte proliferation (27). Second, it remains unclear whether inflammation, or barrier disruption, triggers GPR15L expression in the epidermis in stressed situations *in vivo*, because it is very difficult to distinguish the effect of barrier disruption from subsequent inflammation (or vice versa). This issue is a shared research-associated conundrum in the field of skin barrier and protective response; and hence should be addressed with elegant and elaborate strategies in the future.

In conclusion, we found a new role for GPR15L, the product of a former orphan gene, in regulating proinflammatory response and late differentiation of keratinocytes. We propose that cellular stress, during the breakdown of skin homeostasis, triggers the expression of GPR15L, which contributes to the organization of the protective response and subsequent pathological conditions of the skin. Therefore, our findings suggest that GPR15L production is a key event in various types of skin inflammation and a potential therapeutic target.

## Data Availability Statement

The datasets presented in this study can be found in online repositories. RNA-seq data were deposited in the Gene Expression Omnibus (GEO) database; accession number GSE189751.

## Ethics Statement

All procedures for animal experiments were reviewed and approved by the Animal Research Committee, Graduate School of Medicine, Kyoto University (approval Med Kyo 20532) and performed according to the institutional guidelines. The study using human specimens was approved by the Graduate School of Medicine, Kyoto University (R0743). Written informed consent was obtained from all individuals.

## Author Contributions

TD initiated and supervised the research, obtained funding, performed experiments, and wrote the manuscript. YN, HD, and RM performed the experiments. SatN, VN, RMT, and EK performed the transcriptome analyses. SaeN, TF, EG-Y, and TL predicted GPR15L gene properties. OD, PW, and BR provided methodological support. KK obtained funding and supervised the work. All authors contributed to the article and approved the submitted version.

## Funding

This work was supported by JSPS KAKENHI, grant numbers JP18K08295, JP16K15548 (TD), JP20H05697, and JP15H05790 (KK), and the 8th Rohto Dermatology Prize 2015 (TD).

## Conflict of Interest

The authors declare that the research was conducted in the absence of any commercial or financial relationships that could be construed as a potential conflict of interest.

## Publisher’s Note

All claims expressed in this article are solely those of the authors and do not necessarily represent those of their affiliated organizations, or those of the publisher, the editors and the reviewers. Any product that may be evaluated in this article, or claim that may be made by its manufacturer, is not guaranteed or endorsed by the publisher.

## Supplementary Material

Supplementary Table 1 Primer sequences used for the quantitative RT-PCR analyses.

Supplementary Table 2 DEGs between the *C10orf99*-transfected NHEKs and empty plasmid-transfected NHEKs (false discovery rate: FDR < 0.05).

Supplementary Table 3 The results of database for annotation, visualization, and integrated discovery (DAVID) and Enrichr gene ontology analyses.

Supplementary Table 4 The results of parametric enrichment analysis based on the KEGG functional hierarchy using the GAGE algorithm.

Supplementary Figure 1
**(A)** Quantitative RT-PCR analysis of GPR15L mRNA levels in the skin from newborn mice. Results were normalized to *Gapdh* expression (error bars, SD; n = 3 per group). **(B)** GPR15L protein expression in the upper dermis of lesional skin from a patient with psoriasis. Representative results from the five cases are shown (a scale bar, 20 µm).

Supplementary Figure 2
**(A)** Quantitative RT-PCR analysis of mRNA levels in the epidermis of newborn mice. Results were normalized to *Gapdh* expression (error bars, SD; n = 5 per group). **(B)** Quantitative RT-PCR analysis of IL-1β mRNA levels in mouse primary cultured keratinocytes with indicated stimulations. Results were normalized to *Gapdh* expression (error bars, SD; n = 3 per group).

Supplementary Figure 3 Sample clustering analyses of the RNA-Seq data. **(A)** Hierarchical clustering. **(B)** Multidimensional scaling analysis. **(C)** Principal component analysis (PCA).

## References

[1] Dainichi T Hanakawa S Kabashima K. Classification of Inflammatory Skin Diseases: A Proposal Based on the Disorders of the Three-Layered Defense Systems, Barrier, Innate Immunity and Acquired Immunity. J Dermatol Sci (2014) 76(2):81–9. doi: 10.1016/j.jdermsci.2014.08.010 25242498

[2] Luger T Amagai M Dreno B Dagnelie MA Liao W Kabashima K. Atopic Dermatitis: Role of the Skin Barrier, Environment, Microbiome, and Therapeutic Agents. J Dermatol Sci (2021) 102(3):142–57. doi: 10.1016/j.jdermsci.2021.04.007 34116898

[3] Dainichi T Kitoh A Otsuka A Nakajima S Nomura T Kaplan DH. The Epithelial Immune Microenvironment (EIME) in Atopic Dermatitis and Psoriasis. Nat Immunol (2018) 19(12):1286–98. doi: 10.1038/s41590-018-0256-2 30446754

[4] Matsumoto R Dainichi T Tsuchiya S Nomura T Kitoh A Hayden MS. Epithelial TRAF6 Drives IL-17-Mediated Psoriatic Inflammation. JCI Insight (2018) 3(15):1–14. doi: 10.1172/jci.insight.121175 PMC6129131 30089718

[5] Suply T Hannedouche S Carte N Li J Grosshans B Schaefer M. A Natural Ligand for the Orphan Receptor GPR15 Modulates Lymphocyte Recruitment to Epithelia. Sci Signal (2017) 10(496):1–11. doi: 10.1126/scisignal.aal0180 28900043

[6] Ocon B Pan J Dinh TT Chen W Ballet R Bscheider M. A Mucosal and Cutaneous Chemokine Ligand for the Lymphocyte Chemoattractant Receptor Gpr15. Front Immunol (2017) 8:1111. doi: 10.3389/fimmu.2017.01111 28936214 PMC5594226

[7] Tang T Li L Tang J Li Y Lin WY Martin F. A Mouse Knockout Library for Secreted and Transmembrane Proteins. Nat Biotechnol (2010) 28(7):749–55. doi: 10.1038/nbt.1644 20562862

[8] Yang M Tang M Ma X Yang L He J Peng X. AP-57/C10orf99 is a New Type of Multifunctional Antimicrobial Peptide. Biochem Biophys Res Commun (2015) 457(3):347–52. doi: 10.1016/j.bbrc.2014.12.115 25585381

[9] Pan W Cheng Y Zhang H Liu B Mo X Li T. CSBF/C10orf99, a Novel Potential Cytokine, Inhibits Colon Cancer Cell Growth Through Inducing G1 Arrest. Sci Rep (2014) 4:6812. doi: 10.1038/srep06812 25351403 PMC4212244

[10] Guo P Luo Y Mai G Zhang M Wang G Zhao M. Gene Expression Profile Based Classification Models of Psoriasis. Genomics (2014) 103(1):48–55. doi: 10.1016/j.ygeno.2013.11.001 24239985

[11] Nair RP Duffin KC Helms C Ding J Stuart PE Goldgar D. Genome-Wide Scan Reveals Association of Psoriasis With IL-23 and NF-kappaB Pathways. Nat Genet (2009) 41(2):199–204. doi: 10.1038/ng.311 19169254 PMC2745122

[12] Gudjonsson JE Ding J Johnston A Tejasvi T Guzman AM Nair RP. Assessment of the Psoriatic Transcriptome in a Large Sample: Additional Regulated Genes and Comparisons With *In Vitro* Models. J Invest Dermatol (2010) 130(7):1829–40. doi: 10.1038/jid.2010.36 PMC3128718 20220767

[13] Hueber W Patel DD Dryja T Wright AM Koroleva I Bruin G. Effects of AIN457, a Fully Human Antibody to Interleukin-17A, on Psoriasis, Rheumatoid Arthritis, and Uveitis. Sci Transl Med (2010) 2(52):52ra72. doi: 10.1126/scitranslmed.3001107 20926833

[14] Roberson ED Liu Y Ryan C Joyce CE Duan S Cao L. A Subset of Methylated CpG Sites Differentiate Psoriatic From Normal Skin. J Invest Dermatol (2012) 132(3 Pt 1):583–92. doi: 10.1038/jid.2011.348 PMC3568942 22071477

[15] Swindell WR Johnston A Carbajal S Han G Wohn C Lu J. Genome-Wide Expression Profiling of Five Mouse Models Identifies Similarities and Differences With Human Psoriasis. PloS One (2011) 6(4):e18266. doi: 10.1371/journal.pone.0018266 21483750 PMC3070727

[16] Guttman-Yassky E Suarez-Farinas M Chiricozzi A Nograles KE Shemer A Fuentes-Duculan J. Broad Defects in Epidermal Cornification in Atopic Dermatitis Identified Through Genomic Analysis. J Allergy Clin Immunol (2009) 124(6):1235–44 e58. doi: 10.1016/j.jaci.2009.09.031 20004782

[17] Rodriguez E Baurecht H Wahn AF Kretschmer A Hotze M Zeilinger S. An Integrated Epigenetic and Transcriptomic Analysis Reveals Distinct Tissue-Specific Patterns of DNA Methylation Associated With Atopic Dermatitis. J Invest Dermatol (2014) 134(7):1873–83. doi: 10.1038/jid.2014.87 24739813

[18] Suarez-Farinas M Ungar B Correa da Rosa J Ewald DA Rozenblit M Gonzalez J. RNA Sequencing Atopic Dermatitis Transcriptome Profiling Provides Insights Into Novel Disease Mechanisms With Potential Therapeutic Implications. J Allergy Clin Immunol (2015) 135(5):1218–27. doi: 10.1016/j.jaci.2015.03.003 25840722

[19] Ewald DA Noda S Oliva M Litman T Nakajima S Li X. Major Differences Between Human Atopic Dermatitis and Murine Models, as Determined by Using Global Transcriptomic Profiling. J Allergy Clin Immunol (2017) 139(2):562–71. doi: 10.1016/j.jaci.2016.08.029 27702671

[20] Heiber M Marchese A Nguyen T Heng HH George SR O'Dowd BF. A Novel Human Gene Encoding a G-Protein-Coupled Receptor (GPR15) is Located on Chromosome 3. Genomics (1996) 32(3):462–5. doi: 10.1006/geno.1996.0143 8838812

[21] Deng HK Unutmaz D KewalRamani VN Littman DR. Expression Cloning of New Receptors Used by Simian and Human Immunodeficiency Viruses. Nature (1997) 388(6639):296–300. doi: 10.1038/40894 9230441

[22] Kim SV Xiang WV Kwak C Yang Y Lin XW Ota M. GPR15-Mediated Homing Controls Immune Homeostasis in the Large Intestine Mucosa. Science (2013) 340(6139):1456–9. doi: 10.1126/science.1237013 PMC3762262 23661644

[23] Nguyen LP Pan J Dinh TT Hadeiba H O'Hara E 3rd Ebtikar A. Role and Species-Specific Expression of Colon T Cell Homing Receptor GPR15 in Colitis. Nat Immunol (2015) 16(2):207–13. doi: 10.1038/ni.3079 PMC4338558 25531831

[24] Fischer A Zundler S Atreya R Rath T Voskens C Hirschmann S. Differential Effects of Alpha4beta7 and GPR15 on Homing of Effector and Regulatory T Cells From Patients With UC to the Inflamed Gut In Vivo. Gut (2016) 65(10):1642–64. doi: 10.1136/gutjnl-2015-310022 PMC5036234 26209553

[25] Seong Y Lazarus NH Sutherland L Habtezion A Abramson T He XS. Trafficking Receptor Signatures Define Blood Plasmablasts Responding to Tissue-Specific Immune Challenge. JCI Insight (2017) 2(6):e90233. doi: 10.1172/jci.insight.90233 28352656 PMC5358486

[26] Lahl K Sweere J Pan J Butcher E. Orphan Chemoattractant Receptor GPR15 Mediates Dendritic Epidermal T-Cell Recruitment to the Skin. Eur J Immunol (2014) 44(9):2577–81. doi: 10.1002/eji.201444628 PMC4165750 24838826

[27] Chen C Wu N Duan Q Yang H Wang X Yang P. C10orf99 Contributes to the Development of Psoriasis by Promoting the Proliferation of Keratinocytes. Sci Rep (2018) 8(1):8590. doi: 10.1038/s41598-018-26996-z 29872130 PMC5988722

[28] Sezin T Kempen L Meyne LM Mousavi S Zillikens D Sadik CD. GPR15 is Not Critically Involved in the Regulation of Murine Psoriasiform Dermatitis. J Dermatol Sci (2019) 94(1):196–204. doi: 10.1016/j.jdermsci.2019.01.008 30935778

[29] Nakashima C Otsuka A Kitoh A Honda T Egawa G Nakajima S. Basophils Regulate the Recruitment of Eosinophils in a Murine Model of Irritant Contact Dermatitis. J Allergy Clin Immunol (2014) 134(1):100–7. doi: 10.1016/j.jaci.2014.02.026 24713170

[30] Dainichi T Hayden MS Park SG Oh H Seeley JJ Grinberg-Bleyer Y. PDK1 Is a Regulator of Epidermal Differentiation That Activates and Organizes Asymmetric Cell Division. Cell Rep (2016) 15(8):1615–23. doi: 10.1016/j.celrep.2016.04.051 PMC4909264 27184845

[31] Picelli S Faridani OR Bjorklund AK Winberg G Sagasser S Sandberg R. Full-Length RNA-Seq From Single Cells Using Smart-seq2. Nat Protoc (2014) 9(1):171-81. doi: 10.1038/nprot.2014.006 24385147

[32] Dobin A Davis CA Schlesinger F Drenkow J Zaleski C Jha S. STAR: Ultrafast Universal RNA-Seq Aligner. Bioinformatics (2013) 29(1):15–21. doi: 10.1093/bioinformatics/bts635 23104886 PMC3530905

[33] Liao Y Smyth GK Shi W. Featurecounts: An Efficient General Purpose Program for Assigning Sequence Reads to Genomic Features. Bioinformatics (2014) 30(7):923–30. doi: 10.1093/bioinformatics/btt656 24227677

[34] Harrow J Frankish A Gonzalez JM Tapanari E Diekhans M Kokocinski F. GENCODE: The Reference Human Genome Annotation for The ENCODE Project. Genome Res (2012) 22(9):1760–74. doi: 10.1101/gr.135350.111 PMC3431492 22955987

[35] Robinson MD McCarthy DJ Smyth GK. Edger: A Bioconductor Package for Differential Expression Analysis of Digital Gene Expression Data. Bioinformatics (2010) 26(1):139–40. doi: 10.1093/bioinformatics/btp616 PMC2796818 19910308

[36] Huang da W Sherman BT Lempicki RA. Systematic and Integrative Analysis of Large Gene Lists Using DAVID Bioinformatics Resources. Nat Protoc (2009) 4(1):44–57. doi: 10.1038/nprot.2008.211 19131956

[37] Xie Z Bailey A Kuleshov MV Clarke DJB Evangelista JE Jenkins SL. Gene Set Knowledge Discovery With Enrichr. Curr Protoc (2021) 1(3):e90. doi: 10.1002/cpz1.90 33780170 PMC8152575

[38] Kanehisa M Sato Y Kawashima M Furumichi M Tanabe M. KEGG as a Reference Resource for Gene and Protein Annotation. Nucleic Acids Res (2016) 44(D1):D457–62. doi: 10.1093/nar/gkv1070 PMC4702792 26476454

[39] Luo W Friedman MS Shedden K Hankenson KD Woolf PJ. GAGE: Generally Applicable Gene Set Enrichment for Pathway Analysis. BMC Bioinformatics (2009) 10:161. doi: 10.1186/1471-2105-10-161 19473525 PMC2696452

[40] Uchiyama T Irie M Mori H Kurokawa K Yamada T. FuncTree: Functional Analysis and Visualization for Large-Scale Omics Data. PloS One (2015) 10(5):e0126967. doi: 10.1371/journal.pone.0126967 25974630 PMC4431737

[41] Kawakami E Nakaoka S Ohta T Kitano H. Weighted Enrichment Method for Prediction of Transcription Regulators From Transcriptome and Global Chromatin Immunoprecipitation Data. Nucleic Acids Res (2016) 44(11):5010–21. doi: 10.1093/nar/gkw355 PMC4914117 27131787

[42] Zhang L Tinkle SS. Chemical Activation of Innate and Specific Immunity in Contact Dermatitis. J Invest Dermatol (2000) 115(2):168–76. doi: 10.1046/j.1523-1747.2000.00999.x 10951232

[43] Honda T Egawa G Grabbe S Kabashima K. Update of Immune Events in the Murine Contact Hypersensitivity Model: Toward the Understanding of Allergic Contact Dermatitis. J Invest Dermatol (2013) 133(2):303–15. doi: 10.1038/jid.2012.284 22931926

[44] Iida H Takai T Hirasawa Y Kamijo S Shimura S Ochi H. Epicutaneous Administration of Papain Induces IgE and IgG Responses in a Cysteine Protease Activity-Dependent Manner. Allergol Int (2014) 63(2):219–26. doi: 10.2332/allergolint.13-OA-0621 24662805

[45] van der Fits L Mourits S Voerman JS Kant M Boon L Laman JD. Imiquimod-Induced Psoriasis-Like Skin Inflammation in Mice is Mediated *via* the IL-23/IL-17 Axis. J Immunol (2009) 182(9):5836–45. doi: 10.4049/jimmunol.0802999 19380832

[46] Chiricozzi A Nograles KE Johnson-Huang LM Fuentes-Duculan J Cardinale I Bonifacio KM. IL-17 Induces an Expanded Range of Downstream Genes in Reconstituted Human Epidermis Model. PloS One (2014) 9(2):e90284. doi: 10.1371/journal.pone.0090284 24587313 PMC3938679

[47] Li M Hener P Zhang Z Kato S Metzger D Chambon P. Topical Vitamin D3 and Low-Calcemic Analogs Induce Thymic Stromal Lymphopoietin in Mouse Keratinocytes and Trigger an Atopic Dermatitis. Proc Natl Acad Sci U S A (2006) 103(31):11736–41. doi: 10.1073/pnas.0604575103 PMC1544239 16880407

[48] Motohashi H Katsuoka F Engel JD Yamamoto M. Small Maf Proteins Serve as Transcriptional Cofactors for Keratinocyte Differentiation in the Keap1-Nrf2 Regulatory Pathway. Proc Natl Acad Sci U S A (2004) 101(17):6379–84. doi: 10.1073/pnas.0305902101 PMC404053 15087497

[49] von Scheidt M Zhao Y de Aguiar Vallim TQ Che N Wierer M Seldin MM. Transcription Factor MAFF (MAF Basic Leucine Zipper Transcription Factor F) Regulates an Atherosclerosis Relevant Network Connecting Inflammation and Cholesterol Metabolism. Circulation (2021) 143(18):1809–23. doi: 10.1161/CIRCULATIONAHA.120.050186 PMC8124091 33626882

[50] Yu W Li D Ding X Sun Y Liu Y Cong J. LINC00702 Suppresses Proliferation and Invasion in non-Small Cell Lung Cancer Through Regulating miR-510/PTEN Axis. Aging (Albany NY) (2019) 11(5):1471–85. doi: 10.18632/aging.101846 PMC6428098 30840927

[51] Li T Ren J Ma J Wu J Zhang R Yuan H. LINC00702/miR-4652-3p/ZEB1 Axis Promotes the Progression of Malignant Meningioma Through Activating Wnt/beta-Catenin Pathway. BioMed Pharmacother (2019) 113:108718. doi: 10.1016/j.biopha.2019.108718 30849635

[52] Ghisletti S Barozzi I Mietton F Polletti S De Santa F Venturini E. Identification and Characterization of Enhancers Controlling the Inflammatory Gene Expression Program in Macrophages. Immunity (2010) 32(3):317–28. doi: 10.1016/j.immuni.2010.02.008 20206554

[53] Harada H Fujita T Miyamoto M Kimura Y Maruyama M Furia A. Structurally Similar But Functionally Distinct Factors, IRF-1 and IRF-2, Bind to the Same Regulatory Elements of IFN and IFN-Inducible Genes. Cell (1989) 58(4):729–39. doi: 10.1016/0092-8674(89)90107-4 2475256

[54] Arimoto KI Lochte S Stoner SA Burkart C Zhang Y Miyauchi S. STAT2 is an Essential Adaptor in USP18-Mediated Suppression of Type I Interferon Signaling. Nat Struct Mol Biol (2017) 24(3):279–89. doi: 10.1038/nsmb.3378 PMC5365074 28165510

[55] Peng JC Valouev A Swigut T Zhang J Zhao Y Sidow A. Jarid2/Jumonji Coordinates Control of PRC2 Enzymatic Activity and Target Gene Occupancy in Pluripotent Cells. Cell (2009) 139(7):1290–302. doi: 10.1016/j.cell.2009.12.002 PMC2911953 20064375

[56] Yokoyama C Wang X Briggs MR Admon A Wu J Hua X. SREBP-1, a Basic-Helix-Loop-Helix-Leucine Zipper Protein That Controls Transcription of the Low Density Lipoprotein Receptor Gene. Cell (1993) 75(1):187–97. doi: 10.1016/S0092-8674(05)80095-9 8402897

[57] Cartwright A Schmutz C Askari A Kuiper JH Middleton J. Orphan Receptor GPR15/BOB is Up-Regulated in Rheumatoid Arthritis. Cytokine (2014) 67(2):53–9. doi: 10.1016/j.cyto.2014.02.015 PMC3996549 24725539

[58] Jegodzinski L Sezin T Loser K Mousavi S Zillikens D Sadik CD. The G Protein-Coupled Receptor (GPR) 15 Counteracts Antibody-Mediated Skin Inflammation. Front Immunol (2020) 11:1858. doi: 10.3389/fimmu.2020.01858 32922401 PMC7456807

[59] Foster SR Hauser AS Vedel L Strachan RT Huang XP Gavin AC. Discovery of Human Signaling Systems: Pairing Peptides to G Protein-Coupled Receptors. Cell (2019) 179(4):895–908 e21. doi: 10.1016/j.cell.2019.10.010 31675498 PMC6838683

[60] Barrat FJ Crow MK Ivashkiv LB. Interferon Target-Gene Expression and Epigenomic Signatures in Health and Disease. Nat Immunol (2019) 20(12):1574–83. doi: 10.1038/s41590-019-0466-2 PMC7024546 31745335

[61] Castrillo A Joseph SB Vaidya SA Haberland M Fogelman AM Cheng G. Crosstalk Between LXR and Toll-Like Receptor Signaling Mediates Bacterial and Viral Antagonism of Cholesterol Metabolism. Mol Cell (2003) 12(4):805–16. doi: 10.1016/s1097-2765(03)00384-8 14580333

[62] Ghisletti S Huang W Ogawa S Pascual G Lin ME Willson TM. Parallel SUMOylation-Dependent Pathways Mediate Gene- and Signal-Specific Transrepression by LXRs and PPARgamma. Mol Cell (2007) 25(1):57–70. doi: 10.1016/j.molcel.2006.11.022 17218271 PMC1850387

[63] Gillespie MA Gold ES Ramsey SA Podolsky I Aderem A Ranish JA. An LXR-NCOA5 Gene Regulatory Complex Directs Inflammatory Crosstalk-Dependent Repression of Macrophage Cholesterol Efflux. EMBO J (2015) 34(9):1244–58. doi: 10.15252/embj.201489819 PMC4426483 25755249

[64] Otsuka M Egawa G Dainichi T Okuno T Ishida Y Chow Z. Cutaneous Liver X Receptor Activation Prevents the Formation of Imiquimod-Induced Psoriatic Dermatitis. J Invest Dermatol (2021). doi: 10.1016/j.jid.2021.08.432 34555417

[65] Yoshikawa T Shimano H Amemiya-Kudo M Yahagi N Hasty AH Matsuzaka T. Identification of Liver X Receptor-Retinoid X Receptor as an Activator of the Sterol Regulatory Element-Binding Protein 1c Gene Promoter. Mol Cell Biol (2001) 21(9):2991–3000. doi: 10.1128/MCB.21.9.2991-3000.2001 11287605 PMC86928

[66] Angela M Endo Y Asou HK Yamamoto T Tumes DJ Tokuyama H. Fatty Acid Metabolic Reprogramming *via* mTOR-Mediated Inductions of PPARgamma Directs Early Activation of T Cells. Nat Commun (2016) 7:13683. doi: 10.1038/ncomms13683 27901044 PMC5141517

[67] Dorotea D Koya D Ha H. Recent Insights Into SREBP as a Direct Mediator of Kidney Fibrosis *via* Lipid-Independent Pathways. Front Pharmacol (2020) 11:265. doi: 10.3389/fphar.2020.00265 32256356 PMC7092724

