# Supplementary figures and images for: C10orf99/GPR15L Regulates Proinflammatory Response of Keratinocytes and Barrier Formation of the Skin
Source: Front Immunol. 2022 Feb 22;13:825032. doi: 10.3389/fimmu.2022.825032 (PMC8902463; doi:10.3389/fimmu.2022.825032)

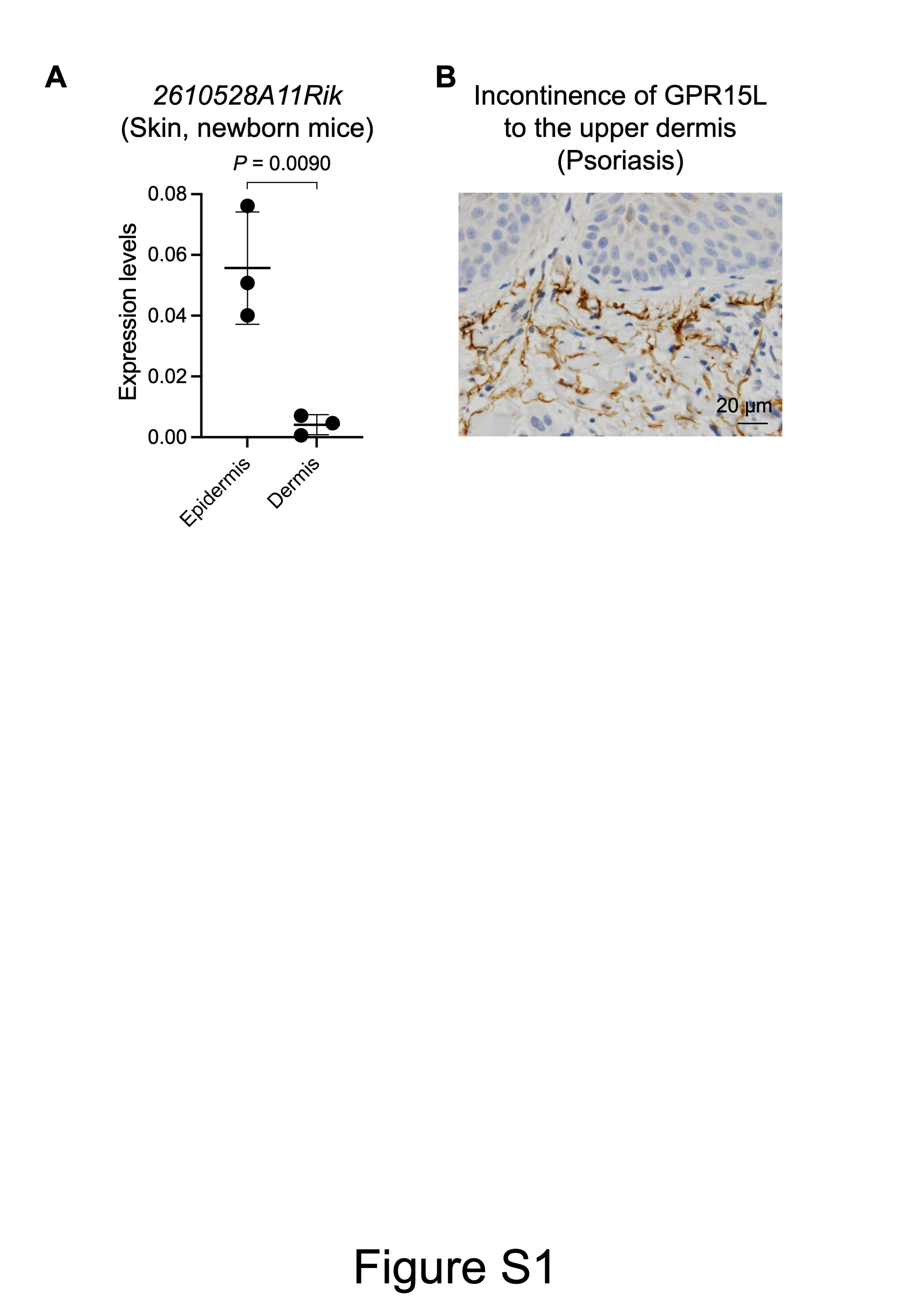

Supplement: Supplementary Figure 1 — (A) Quantitative RT-PCR analysis of GPR15L mRNA levels in the skin from newborn mice. Results were normalized to Gapdh expression (error bars, SD; n = 3 per group). (B) GPR15L protein expression in the upper dermis of lesional skin from a patient with psoriasis. Representative results from the five cases are shown (a scale bar, 20 µm). [file Image_1.tiff]

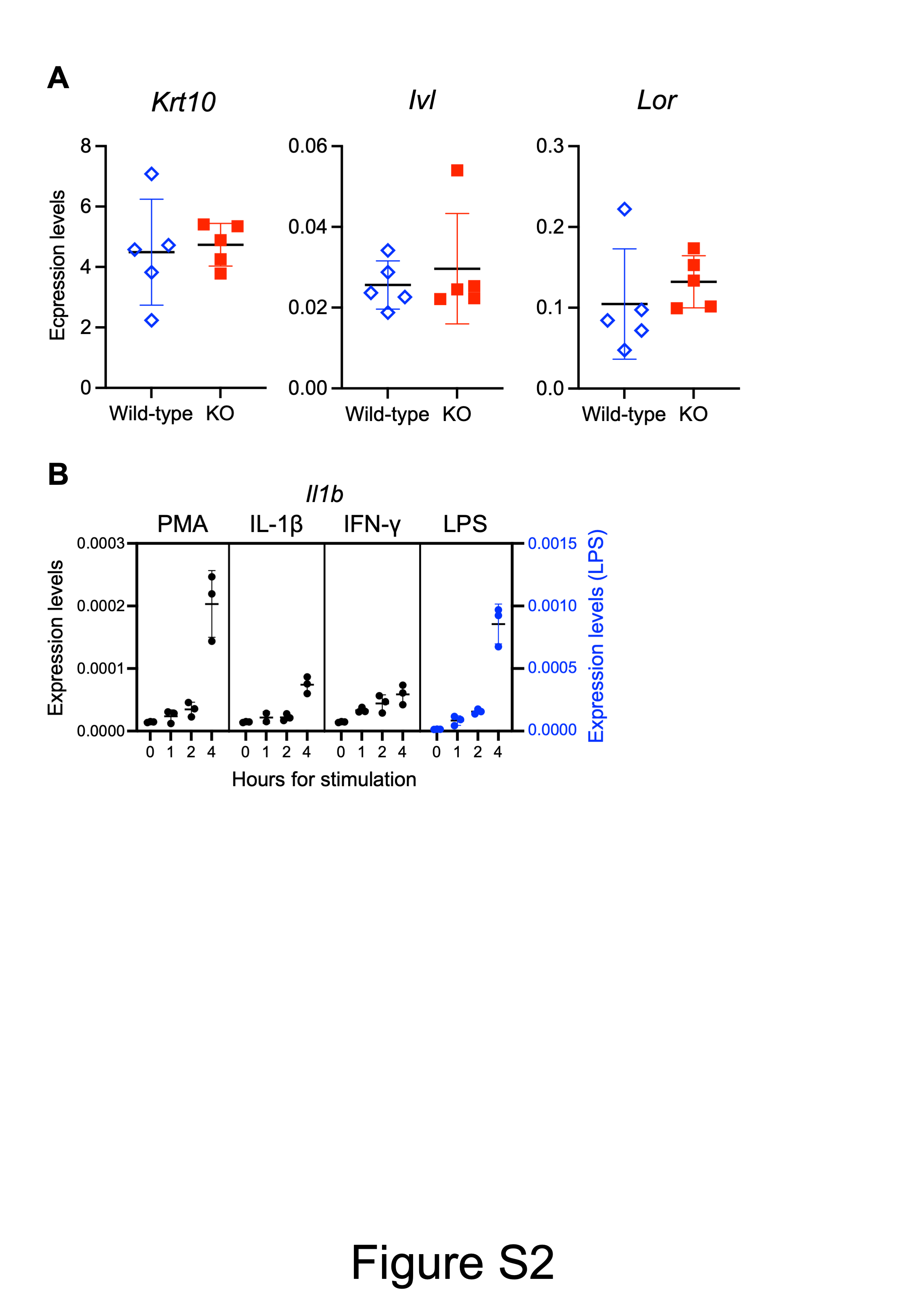

Supplement: Supplementary Figure 2 — (A) Quantitative RT-PCR analysis of mRNA levels in the epidermis of newborn mice. Results were normalized to Gapdh expression (error bars, SD; n = 5 per group). (B) Quantitative RT-PCR analysis of IL-1β mRNA levels in mouse primary cultured keratinocytes with indicated stimulations. Results were normalized to Gapdh expression (error bars, SD; n = 3 per group). [file Image_2.tiff]

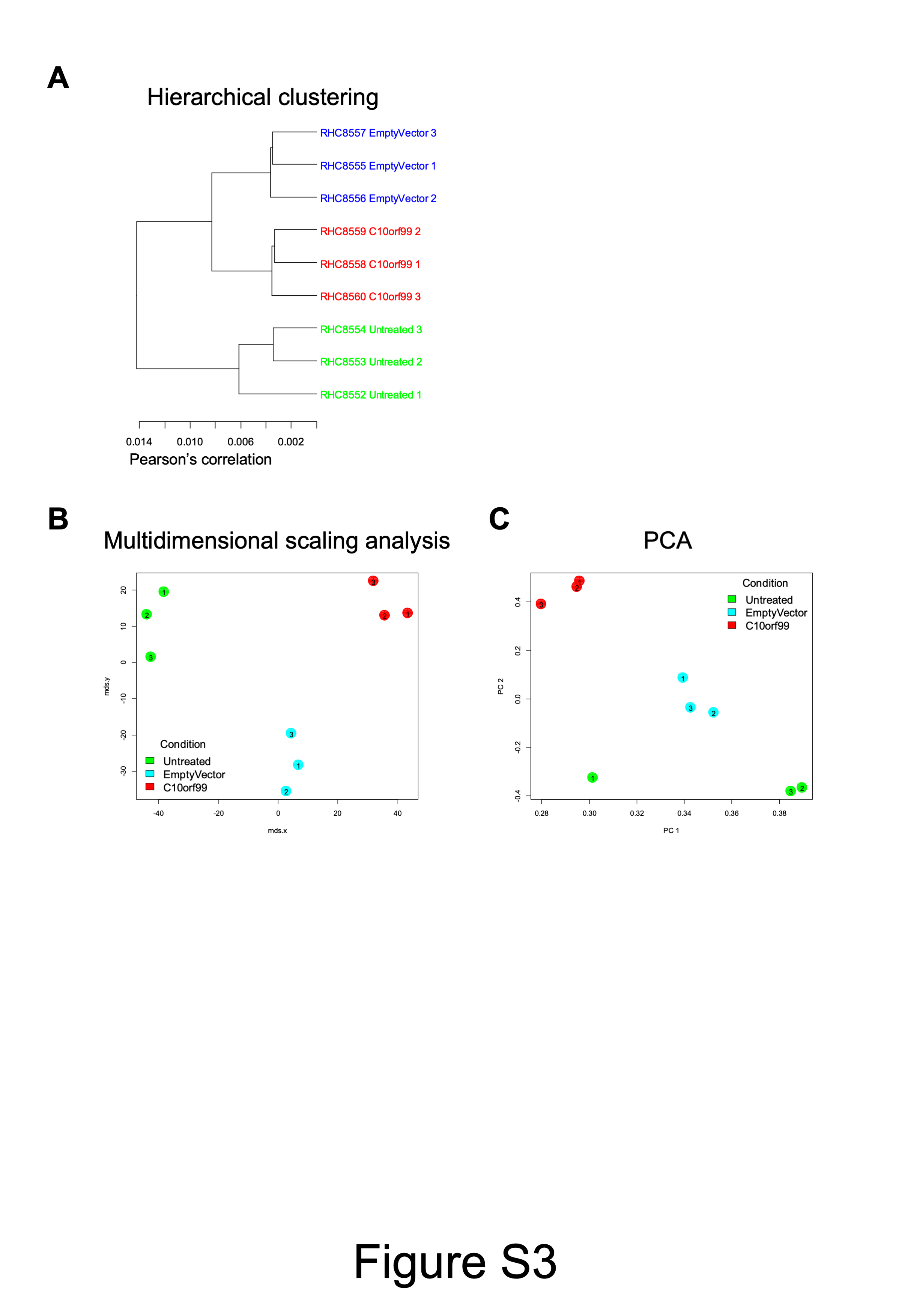

Supplement: Supplementary Figure 3 — Sample clustering analyses of the RNA-Seq data. (A) Hierarchical clustering. (B) Multidimensional scaling analysis. (C) Principal component analysis (PCA). [file Image_3.tiff]
